# Syncytium cell growth increases Kir2.1 contribution in human iPSC-cardiomyocytes

**DOI:** 10.1152/ajpheart.00148.2020

**Published:** 2020-09-28

**Authors:** Weizhen Li, Julie L. Han, Emilia Entcheva

**Affiliations:** Department of Biomedical Engineering, George Washington University, Washington, District of Columbia

**Keywords:** all-optical electrophysiology, cardiac electrophysiology, cell density, human iPSC-cardiomyocytes, *I*_k1_, Kir2.1, maturity, optogenetics

## Abstract

Human induced pluripotent stem cell-derived cardiomyocytes (hiPSC-CMs) enable cardiotoxicity testing and personalized medicine. However, their maturity is of concern, including relatively depolarized resting membrane potential and more spontaneous activity compared with adult cardiomyocytes, implicating low or lacking inward rectifier potassium current (*I*_k1_). Here, protein quantification confirms Kir2.1 expression in hiPSC-CM syncytia, albeit several times lower than in adult heart tissue. We find that hiPSC-CM culture density influences Kir2.1 expression at the mRNA level (potassium inwardly rectifying channel subfamily J member 2) and at the protein level and its associated electrophysiology phenotype. Namely, all-optical cardiac electrophysiology and pharmacological treatments reveal reduction of spontaneous and irregular activity and increase in action potential upstroke in denser cultures. Blocking *I*_k1_-like currents with BaCl_2_ increased spontaneous frequency and blunted action potential upstrokes during pacing in a dose-dependent manner only in the highest-density cultures, in line with *I*_k1_’s role in regulating the resting membrane potential. Our results emphasize the importance of syncytial growth of hiPSC-CMs for more physiologically relevant phenotype and the power of all-optical electrophysiology to study cardiomyocytes in their multicellular setting.

**NEW & NOTEWORTHY** We identify cell culture density and cell-cell contact as an important factor in determining the expression of a key ion channel at the transcriptional and the protein levels, KCNJ2/Kir2.1, and its contribution to the electrophysiology of human induced pluripotent stem cell-derived cardiomyocytes. Our results indicate that studies on isolated cells, out of tissue context, may underestimate the cellular ion channel properties being characterized.

## INTRODUCTION

The growing use of human induced pluripotent stem cell-derived cardiomyocytes (hiPSC-CMs) provides genetically diverse human cardiac models to study cardiac arrhythmia patients with different phenotypes and to assess proarrhythmic risk of new drugs in vitro (36, 39). Currently, several cardiomyopathy phenotypes have been successfully recapitulated in vitro, including dilated cardiomyopathy (40), hypertrophic cardiomyopathy (26), and arrhythmogenic right ventricle cardiomyopathy (5). Furthermore, these experimental cardiac models are used for discovering new drug therapies (46) and for cardiotoxicity screening (13). This approach overcomes the limitations of species differences in cardiac electrophysiology when animal models are used to tackle human diseases (8) and the limitations of studying ion channel macromolecular structures in artificial heterologous expression systems (17). As the main source of healthy human cardiomyocytes, hiPSC-CMs have also been widely used in basic science investigations of human cardiomyocyte calcium handling, metabolism studies, single-cell and multicellular contractility, and others (29).

Outstanding concerns about hiPSC-CMs are related to their level of maturity compared with adult cardiac tissue (13). Differences from primary adult cardiomyocytes are seen in cell morphology, calcium handling, glycolysis-based metabolism, and higher cell automaticity (29). Although spontaneous contractions of hiPSC-CMs are considered a sign of successful differentiation, high-frequency spontaneous activity is also a proarrhythmic trait in ventricular cells, and it influences the hiPSC-CM’s credibility as a proper in vitro model. It has been shown that the spontaneous activity of hiPSC-CMs may be partially influenced by low levels of the inward rectifier potassium current (*I*_k1_) (27, 42). *I*_k1_, encoded primarily by KCNJ2 (potassium inwardly rectifying channel subfamily J member 2) and conducted through the Kir2.1-formed pore, plays an important role in the stabilization of the cardiac resting potential, which influences the upstroke of the action potential and potentially the late repolarization of the action potential. Insufficient number or low conductance of functional Kir2.1 ion channels can cause lower membrane resistance (37), with a net inward current during rest, thus a relatively depolarized resting membrane potential, increased spontaneous rates (33), and slower upstroke (through the effects of the resting membrane potential on sodium channel availability) and potentially can also affect the action potential duration of hiPSC-CMs.

Several approaches have been pursued to specifically increase *I*_k1_ in hiPSC-CMs, i.e., *1*) electrical injection of calculated *I*_k1_-like current via dynamic voltage clamp was applied (4, 31); *2*) optical dynamic clamp (ODC) has been applied using light and outward-current-generating opsin ArchT to create a computationally identical *I*_k1_ contribution (35); and *3*) adenoviral overexpression of Kir2.1 has been applied to boost *I*_k1_ in hiPSC-CMs (27, 42). Through increased functional contribution of *I*_k1_ (or *I*_k1_-like current), all three methods, along with other general maturation strategies (15), have claimed a more mature-looking action potential with less depolarized resting membrane potential and suppressed spontaneous activity. As a result, more physiologically relevant responses to ion channel blockers were reported.

Standard studies of ion channel contributions, including all *I*_k1_ studies in hiPSC-CMs, are conducted on single cells using voltage-clamp technology. Although these studies offer the only direct way of quantifying *I*_k1_, they come with limitations, as isolated cardiomyocytes are phenotypically different from cells that exist in a well-coupled cardiac tissue. The cell viability at the end of the cell isolation and the small number of cells that can be studied by such manual techniques may induce selection bias (17). Previous studies (10, 19, 47) have drawn attention to the higher variability in cell morphology and function, including action potential morphology, exhibited by hiPSC-CMs cultured at low plating density. Other reports have indicated possible effects of cell culture density and cell-cell contacts on gene transcription of specific ion channels (16, 37, 41, 47). These studies prompted us to consider that hiPSC-CM multicellular growth conditions may influence cell maturity and the contributions of *I*_k1_-like current to cardiac cell function.

**Fig. 1.**
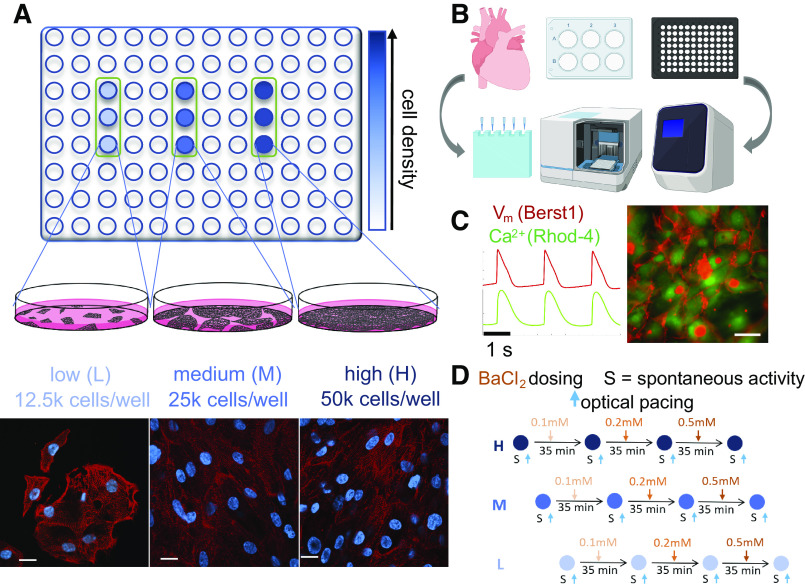
Experimental design using KCNJ2/Kir2.1 quantification and all-optical electrophysiology to probe the effects of *I*_k1_-like current contributions in syncytia of human iPSC-CMs. *A*: 96-well plate format was used for functional tests. High (H), medium (M), and low (L) cell densities were achieved by plating: 50,000, 25,000, and 12,500 cells per well, respectively. Shown are example immunofluorescence images from the 3 density groups, using α-actinin (red) and nuclear DAPI labeling (blue). Scale bar, 20 μm. *B*: protein quantification for human adult heart tissue samples, rat heart, and human iPSC-CMs grown in 6-well plates was done using standard Western blot, while 96-well format small samples of hiPSC-CMs at 2 densities were processed using capillary-based Western blot (Wes by ProteinSimple); Cells-to-CT by Invitrogen was used for quantification of mRNA by qPCR. *B* was created with BioRender.com. *C*: functional measurements were done using all-optical electrophysiology. Example traces and image of a dual-labeled sample for voltage and calcium recordings are shown. Scale bar, 50 μm. *D*: pharmacological probing for *I*_k1_ contributions by sequential application of increasing doses of BaCl_2_. Functional (voltage and calcium) data were simultaneously collected 35 min after each dose treatment under spontaneous conditions (S) and under optical pacing. *I*_k1_, inward rectifier potassium current; hiPSC-CM, human induced pluripotent stem cell-derived cardiomyocytes; KCNJ2, potassium inwardly rectifying channel subfamily J member 2.

Several voltage-clamp studies of isolated myocytes from adult human heart tissue have reported *I*_k1_ values between 3.6 and 32 pA/pF at highly hyperpolarized potentials of −100 mV, whereas values for hiPSC-CMs were found to be 10 to 100 times lower (31), although recent studies question these findings (18). At these hyperpolarized voltage levels, *I*_k1_ is an inward current and indicates more the power to excite rather than maintain resting membrane potential. The peak outward *I*_k1_ for isolated myocytes from adult human heart tissue has been reported in the range of 0.4 to 2.2 pA/pF (9, 31); the values for the outward *I*_k1_ in hiPSC-CMs have been controversial. Through computations and dynamic clamp studies, it has been shown that peak outward *I*_k1_ influences the resting membrane potential for values under ∼3 pA/pF, i.e., within the physiological range. To this end, protein quantification and direct comparison between multicellular hiPSC-CMs and adult human ventricle has not been done. Furthermore, with a few exceptions, detailed functional interrogations of *I*_k1_’s contributions in multicellular hiPSC-CMs are mostly lacking. *I*n this study, we investigate the role of cell density on KCNJ2/Kir2.1 and related hiPSC-CMs electrophysiology (Fig. 1) using mRNA and protein quantification, all-optical cardiac electrophysiology, pharmacological probing, and computational analysis.

## METHODS

### 

#### Human iPSC-CM plating and cell culture.

Human iPSC-derived cardiomyocytes (the standard iCell Cardiomyocytes^2^ CMC-100-012-001 from a female Caucasian donor, and a Mycell hiPSC-CM-1X 01395 line from a male donor) from Fujifilm Cellular Dynamics International (CDI) were thawed based on the manufacturer’s instructions. For standard Western Blot protein quantification, iCell iPSC-CMs were plated on fibronectin-coated six-well plate with recommended plating density of 156,000 cells/cm^2^.

For gene/protein quantification in small samples (96-well format), newer techniques were adopted to run qPCR and Western blotting. For these, female (iCell) and male (MyCell) cells from the two cell lines were plated in fibronectin-coated 96-well plates at two densities: 50,000 cells/well (high density) and 18,500 cells/well (medium to low density). These were then processed for either qPCR (using Cells-to-C_T_ by Invitrogen) or for capillary-based Western blot (WES by ProteinSimple), as described below.

For functional recordings, iCell Cardiomyocytes^2^ were plated on fibronectin-coated 96-well glass-bottom plates. Three tested cell culture densities were 156,000 cells/cm^2^, 78,000 cells/cm^2^, and 39,000 cells/cm^2^, which represented 50,000 (high-density), 25,000 (medium-density) or 12,500 (low-density) cells per well, respectively. All functional studies were conducted on *days 7–8* after thaw.

#### Optogenetic transduction of hiPSC-CMs.

To enable optical pacing, hiPSC-CMs were infected with Ad-CMV-hChR2(H134R)-EYFP 5 days after cell plating, as previously described (2, 3). Maintenance medium, containing viral doses of multiplicity of infection (MOI 350), was applied for 2 h. In the 2-h period, cells were incubated under 37 °C and 5% CO_2_ and gently agitated every 20 min. After 2 h of infection, normal maintenance medium was applied. Functional tests were performed 48 h after viral delivery.

#### hiPSC-CM protein collection for standard Western blot analysis.

Protein samples from hiPSC-CMs were collected 5 days after cell plating. The whole process was done on ice. After ice-cold PBS washes, 120 μL of RIPA buffer was added in each well, and the plate was shaken for 5 min. For the low-density group, two wells were collected into 120 μL of RIPA buffer to get a higher protein concentration. After cells had detached from plate, suspended cell content was transferred into microcentrifuge tubes and spun at 15,000 *g* for 30 min in a precooled 4°C centrifuge. Clear supernatant was separated for protein quantification.

#### Adult human heart tissue and rat tissue processing for standard Western blot analysis.

Flash-frozen adult human heart tissue was purchased from Amsbio and stored at −80°C. Healthy adult rat and 4-day-old neonatal rats were euthanized for ventricular tissue collection. Adult rat heart was cannulated with a 50-mL syringe and perfused with 1× PBS before dissection. The dissected tissues were put in 1.5-mL microcentrifuge tubes and put in liquid nitrogen to flash-freeze until no bubble was generated. Neonatal rat hearts were washed in 1× PBS and flash-frozen in 1.5-mL microcentrifuge tubes until no bubble was generated. Samples were stored at −80°C.

For protein collection, 30 mg of frozen tissue was minced and put on a TissueLyser with metal beads and 1 mL of RIPA buffer (Thermo Fisher Scientific) and run at 50 Hz for 2 min. Tissue lysis then was spun on a 4°C precooled centrifuge for 1 min to remove the foam, and then the TissueLyser step was repeated until tissue was no longer visible: a total of 30-min centrifugation at 14,000 *g* at 4°C.

#### Protein quantification (Kir2.1) using standard Western blot analysis.

The total protein amount was quantified using standard BCA assay in all lysates. Then 5–10 μg of protein per sample was loaded onto 4–20% gradient gel (Bio-Rad) and the gel was run for 2 h at 90 mV. Then, bands were transferred onto nitrocellulose membrane using a semidry transfer system (Bio-Rad). Nonfat milk (5%, Nestle) in TBST buffer (Bio-Rad) was used to block the membrane for 1 h in room temperature. For probing, Kir2.1 antibody (Abcam, ab65796) was diluted 1:200 in 5% nonfat milk in TBS-Tween (TBST) and incubated with the nitrocellulose membrane at 4°C overnight. The membrane was rinsed with TBST three times, 10 min each. Horseradish peroxidase (HRP)-goat anti-rabbit antibody (Abcam, ab6721) diluted 1:1,000 in 5% nonfat milk in TBST was applied for 1 h under room temperature. After three 10-min TBST washes, the HRP signal was enhanced by Radiance Plus (Azure) for imaging. Images were taken by Azure C600 Biosystem. After stripping of the membrane with striping buffer (15 g glycine, 1 g SDS, and 10 mL Tween 20 in 1 liter of ultrapure water, pH adjusted 2.2), the membrane was blocked with 5% nonfat milk in TBST again for 1 h at room temperature. GAPDH (Abcam, ab181602) was probed as loading control; 1:1,000 GAPDH antibody in 5% nonfat milk in TBST was incubated overnight at 4°C. The same secondary antibody was used to probe the bands, and blots were imaged with the same method.

#### Gene expression (KCNJ2) quantification in hiPSC-CMs in 96-well format.

To quantify mRNA using the small number of cells in the 96-well format, we adopted the Power SYBR Green Cells-to-C_T_ Kit (Invitrogen) and applied the method to both cell lines. Cells from 96-well format were harvested per the manufacturer’s instructions and pooled as follows: high-density samples (50,000/well) from individual wells were run as independent qPCR samples, while the lower-density (18,500/well) samples were pooled from two wells into independent qPCR samples. Technical triplicates were run for each independent sample. qPCR analysis was performed on a QuantStudio 3 Real-Time PCR System (Thermo Fisher Scientific) with QuantStudio Design and Analysis Software (Thermo Fisher Scientific). KCNJ2 gene expression was normalized to the expression of housekeeping gene GAPDH (primers for KCNJ2: forward GTGCGAACCAACCGCTACA, reverse CCAGCGAATGTCCACACAC; primers: for GAPDH: forward GGAGCGAGATCCCTCCAAAAT, reverse GGCTGTTGTCATACTTCTCATGG), using the standard ΔΔC_T_ method.

#### Protein quantification (Kir2.1) by Wes: capillary Western blot in hiPSC-CMs in 96-well format.

For small protein samples, such as the ones from 96-well format used for functional measurements, we applied Wes ProteinSimple to be able to get a signal (standard gel-based Western blot requires much more protein). The results from this capillary-based system can be displayed as pseudobands. Cell lysates were loaded onto a microplate following the user manual. Each higher-density sample was used directly to constitute an independent sample, whereas lower-density samples were pooled from four wells for each sample. Kir2.1 (Abcam ab65796) and GAPDH (Abcam ab181602) were diluted 1:10 and 1:4,000, respectively, with Antibody Diluent 2 (ProteinSimple). Anti-rabbit detection module for Wes (ProteinSimple) was used perthe manufacturer’s suggestions. Compass for SW software was used for system setting and data processing. Sample separation time, antibody diluent time, primary antibody time, and secondary antibody time were set to 30 min each. Separation voltage was 375 V.

#### Functional measurements with all-optical cardiac electrophysiology.

Functional experiments were performed using all-optical cardiac electrophysiology as described earlier (22, 23). These were carried out at room temperature in Tyrode's solution (in mM: NaCl, 135; MgCl_2_, 1; KCl, 5.4; CaCl_2_, 2; NaH_2_PO_4_, 0.33; glucose, 5.1; and HEPES, 5, adjusted to pH 7.4 with NaOH). For functional data acquiring and analysis, three regions in each well were chosen randomly. Cell clusters in middle- and low-density groups were chosen without specified preassumption. Regions were chosen far enough apart to not image one area twice. The optical setup was built around an inverted microscope (Nikon Eclipse Ti2), as described (22, 23). Optical pacing was done by 470-nm light pulses of 5 ms at 0.6–0.8 Hz, using irradiances of 0.4–2 mW·mm^2^, as needed. Simultaneous voltage-calcium measurements were performed using temporal multiplexing and iXon Ultra 897 electron-multiplying charge coupled device camera (Andor Technology Ltd., Belfast, UK), run at 200 fps and covering a field of view of ∼400 × 400 μm. For spectral compatibility with channelrhodopsin-2 (*ChR2*), we used a calcium dye rhodamine 4 acetoxy methyl ester (Rhod-4 AM) at 10 μM (AAT Bioquest, Sunnyvale, CA) with fluorescence excitation and emission peaks at 530  and 605 nm, respectively, and a near-infrared voltage dye BeRST1 at 1 μM (from Evan W. Miller, University of California, Berkeley) with fluorescence excitation at 660 nm and emission above 680 nm, as described (23). Signals were filtered and analyzed using an automated in-house software developed to extract relevant parameters related to the action potentials and calcium transients under spontaneous and optically paced conditions (22, 23).

#### Blocking I_k1_-like current with barium chloride.

Different doses of 0.1, 0.2, and 0.5 mM barium chloride (BaCl_2_, Sigma-Aldrich) were applied on each sample serially with 35-min intervals in-between. All-optical electrophysiology was applied to capture spontaneous and optically paced voltage and calcium from multiple locations after each 35-min treatment. The selected doses of BaCl_2_ for relatively selective suppression of *I*_k1_ were based on prior studies that used 0.1 to 0.5 mM (for up to 100% suppression) specifically in hiPSC-CMs (6, 9, 27, 42).

#### Blocking calcium-dependent pathways with SEA400 and ryanodine.

Suppression of spontaneous calcium release was done by blocking the forward mode of the Na^+^/Ca^2+^ exchanger (NCX) using SEA0400 (ApexBio, cat. no. A3811) at 2 μM and at 4 μM and by blocking the ryanodyne receptors (RYR) using ryanodine (Tocris, cat. no. 1329) at 10 μM. All-optical electrophysiology was applied to capture spontaneous calcium activity in the low- and high-density samples before and 5 min after drug application.

#### Blocking clathrin-dependent protein degradation with dynasore.

Dynasore (Sigma-Aldrich, cat. no. D7693) was dissolved in DMSO at 10 mM and stored at −20°C. Upon usage, it was diluted in cell culture medium at 10 µM applied for 24 or 48 h, based on prior studies (43). After the treatment, cells were lysed for protein quantification using RIPA buffer.

#### Computational modeling of the I*_k1_* contribution to action potential parameters.

Action potential simulations were done using the Luo Rudy’s mammalian ventricular cell mathematical model (28), which includes the main factors that shape the action potential morphology. In the modeling, the *I*_k1_ current density was varied under S1–S2 stimulation protocol. The measured outputs included action potential upstroke velocity and action potential duration at 90% repolarization (APD_90_).

#### Statistical analysis.

Comparisons between different groups were done using either one-way or two-way ANOVA with a Tukey–Kramer post hoc correction for multiple comparisons. Significant differences were considered at *P* < 0.05.

## RESULTS

### 

#### Syncytia of hiPSC-CMs express Kir2.1, albeit at lower levels than adult human tissue; blocking degradation pathways does not rescue Kir2.1 levels.

**Fig. 2.**
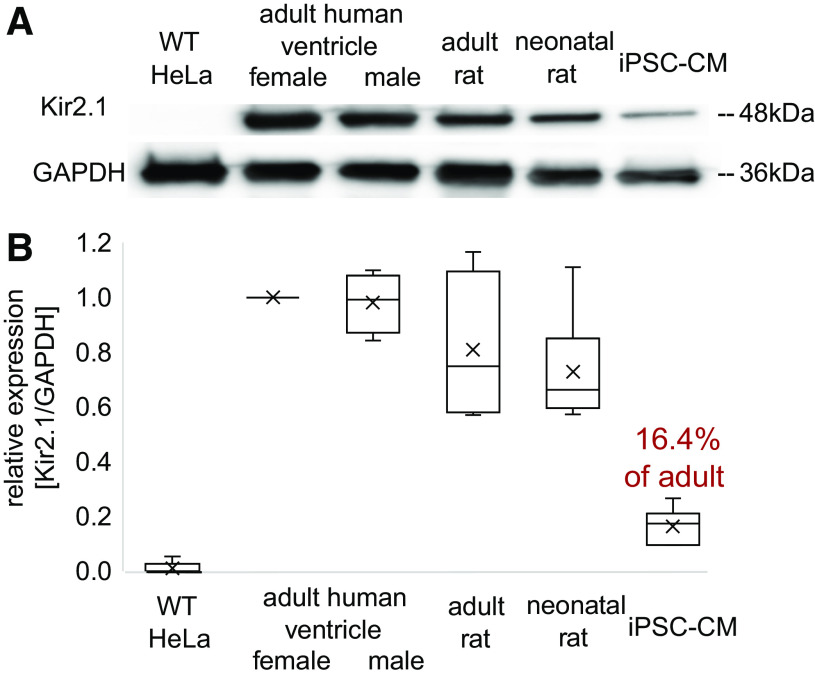
Protein quantification of Kir2.1 in human induced pluripotent stem cell-derived cardiomyocytes (iPSC-CMs), adult human ventricle, adult and neonatal rat ventricular tissue, and wild-type HeLa cells as a negative control. *A*: Western blot results for Kir2.1 protein expression and GAPDH as a loading control. Kir2.1 protein is detected at around 48 kDa. *B*: Western blot quantification based on normalized Kir2.1/GAPDH ratio (*n* = 4–9 per sample type, data pooled from 5 different runs). Tissue samples came from female and male adult human left ventricle as *n* = 1 biological replicate per sex, with *n* = 5 and 4 technical replicates, neonatal and adult rat hearts as *n* = 2, and 1 biological replicate with *n* = 8 and 4 technical replicates; iPSC-CM samples as *n* = 2 biological replicates with *n* = 9 technical replicates came from different cell cultures. Data were normalized by the values for the female human ventricle (which was included as a sample in all 5 runs) and shown as box-whisker plot.

Previous voltage-clamp studies in isolated hiPSC-CMs have reported the lack of or insufficient levels of *I*_k1_ compared with adult cardiomyocytes (31), with differences at the single-cell level on the order of 10–100 times. Here, we quantified Kir2.1 protein levels in hiPSC-CM syncytia by using standard Western blot and compared those with levels found in various cardiac tissues, including adult human ventricular tissue and adult and neonatal rat ventricular tissues, using wild-type HeLa cells as a negative control (Fig. 2). Kir2.1 protein was detected at around ∼48 kDa in all samples except wild-type HeLa cells. The neonatal and the adult rat heart ventricles showed similar robust expression, which was slightly less than the human hearts. This result is in line with studies reporting presence of *I*_k1_ in the rat ventricle and little change after postnatal day 5 (20, 30, 45).

The hiPSC-CM syncytia were found to have lower normalized protein levels (based on Kir2.1/GAPDH ratio) compared with human heart and rat heart samples. Based on these results (*n* = 4–9 per group), hiPSC-CMs’ Kir2.1 expression was about sixfold lower (or ∼16.4%) compared with adult human ventricular tissue (Fig. 2*B*). We confirm previously observed lower Kir2.1 levels in hiPSC-CMs; however, we show less dramatic differences at the protein level (by an order of magnitude –6-fold vs. up to 100-fold) compared with direct measurements of *I*_k1_ using voltage-clamp in single cells. These findings drew our attention to the possibility that syncytial growth might influence the contribution of *I*_k1_ to electrophysiological function in important ways and motivated the pursuit of functional experiments in various hiPSC-CMs’ growth condition and changing cell culture density.

To check the possibility that abnormally fast degradation might be contributing to the lower Kir2.1 protein levels in hiPSC-CMs, we applied pharmacological blocking of lysosomal degradation of Kir2.1 (Supplemental Fig. S1; supplemental material is found at https://doi.org/10.6084/m9.figshare.12940010).

A previous study (43) has demonstrated that dynasore, a noncompetitive inhibitor of GTPase activity of dynamin, can inhibit the clathrin-mediated endocytosis of Kir2.1 and preserve it from degradation. We treated syncytial samples of hiPSC-CMs with 10 µM dynasore over 24 h and saw only mild effects on Kir2.1 protein levels (Supplemental Fig. S1).

#### KCNJ2 gene expression and Kir2.1 protein levels increase in denser cultures of hiPSC-CMs.

Three cell density conditions were established by plating 50,000 (high), 25,000 (medium) and 12,500 cells (low) per well (Fig. 1*A*). Under the high-density condition, well-connected syncytium was formed, with synchronous contractions. Middle-density wells formed less connected cell subgroups with gaps in between. Cells in low-density wells were more separated, forming smaller groups (Supplemental Fig. S2) and more likely to exhibit asynchronous subgroup behavior, as seen in other studies as well (21).

**Fig. 3.**
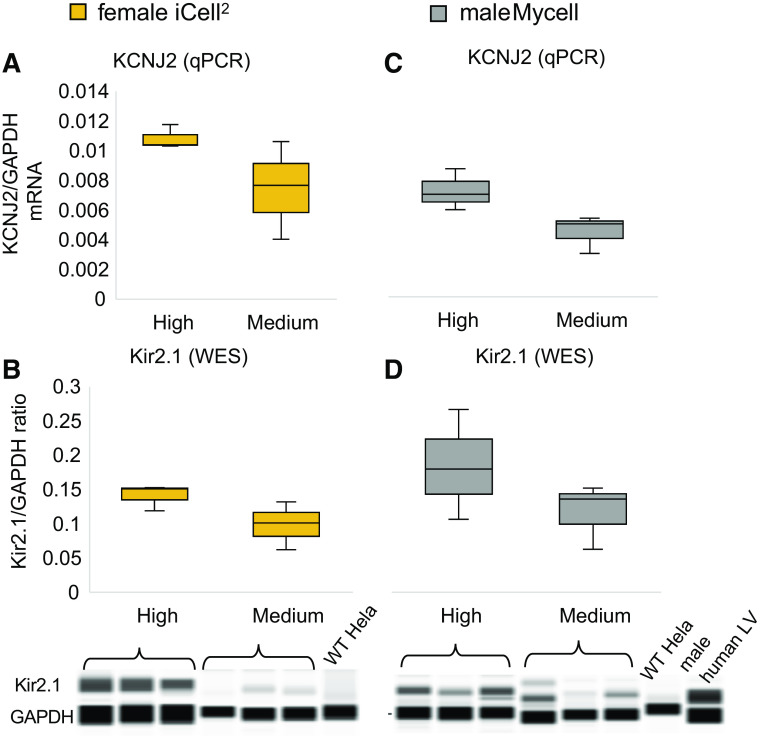
Gene expression [KCNJ2 (potassium inwardly rectifying channel subfamily J member 2)] and protein expression (Kir2.1) are reduced in human induced pluripotent stem cell-derived cardiomyocytes (hiPSC-CMs) cultured at lower plating densities for female and male hiPSC-CMs. Gene expression of KCNJ2 (*A* and *C*) and protein expression of Kir2.1 (*B* and *D*) were quantified in high-density (50,000 cells/well) and low- to medium-density (18,500 cells/well) of hiPSC-CM cultures in a 96-well format, using qPCR and Wes, respectively. Normalization was done by gene or protein levels of GAPDH in the same sample. Female hiPSC-CMs (the standard iCell2), orange, and male donor hiPSC-CMs (MyCell iPSC-CM-1X 01395 donor), gray, were used. qPCR data are presented as *n* = 3 biological replicates with *n* = 3 technical replicates per density and sex. WES data are presented as *n* = 3 biological replicates per density and sex. Male and female cell samples were done in separate runs. For WES controls, wild-type (WT) HeLa cells were used as a negative control and human left ventricle (LV) as a positive control for Kir2.1. Analysis of variance yielded significant differences in the qPCR data due to cell density and due to sex, whereas the protein data did not reach significance but followed a similar trend.

Considering that low levels of *I*_k1_ are a key driver of spontaneous oscillations in ventricular cells (33, 37, 38), we sought to quantify KCNJ2 gene transcription and Kir2.1 protein expression under different density conditions (Fig. 3). Two human iPSC-CM lines, the standard iCell Cardiomyocytes^2^ (female) and Mycell (01395 male donor) were tested in parallel. In replicating the 96-well format of the experiments, we could not get enough material to run standard qPCR and Western blot. Instead, we used Cells-to-CT (Invitrogen) for qPCR, Fig. 3, *A* and *C* and a capillary-based protein quantification technique (Wes by ProteinSimple), Fig. 3, *B* and *D*, suitable for working with small samples and offering picogram sensitivity. We compared GAPDH-normalized KCNJ2 gene expression and Kir2.1 protein levels between high (50,000 cells/well) and medium- to low- (18,500 cells/well) density hiPSC-CM plated in 96-wells. In the Wes protein quantification, antibody multiplexing was optimized for Kir2.1 (1:10) and GAPDH (1:4,000), which enabled signal normalization. The qPCR data (KCNJ2/GAPDH ratio) indicated 31 and 39% lower KCNJ2 expression for the female (iCell) and male (Mycell) iPSC-CMs, respectively, when culture density was reduced by 60%. Quantified Kir2.1/GAPDH protein ratios suggested 30 and 37% lower Kir2.1 levels for female and male iPSC-CMs, respectively, when culture density was reduced by 60% (*n* = 3 per sex and case). For the qPCR data in Fig. 3, *A* and * C*, two-way ANOVA with Tukey–Kramer post hoc correction indicated a significant difference in KCNJ2 expression between the two cell density cultures (*P* = 0.026) and between the sexes (*P* = 0.011). We note that the male and female runs were not done at the same time, so the sex differences may simply reflect different run conditions rather than true differences. However, the comparison between cell densities was conducted with technical replicates within the same run (for each sex) and indicates a significant effect of plating density on KCNJ2. Two-way ANOVA on the protein expression data did not reach significant differences as a function of sex or density despite similar trends to the mRNA data.

#### Frequency of spontaneous oscillations in hiPSC-CMs decreases with higher cell density; pharmacological probing confirmed functional Kir2.1 contribution to pacemaking in denser cell cultures.

**Fig. 4.**
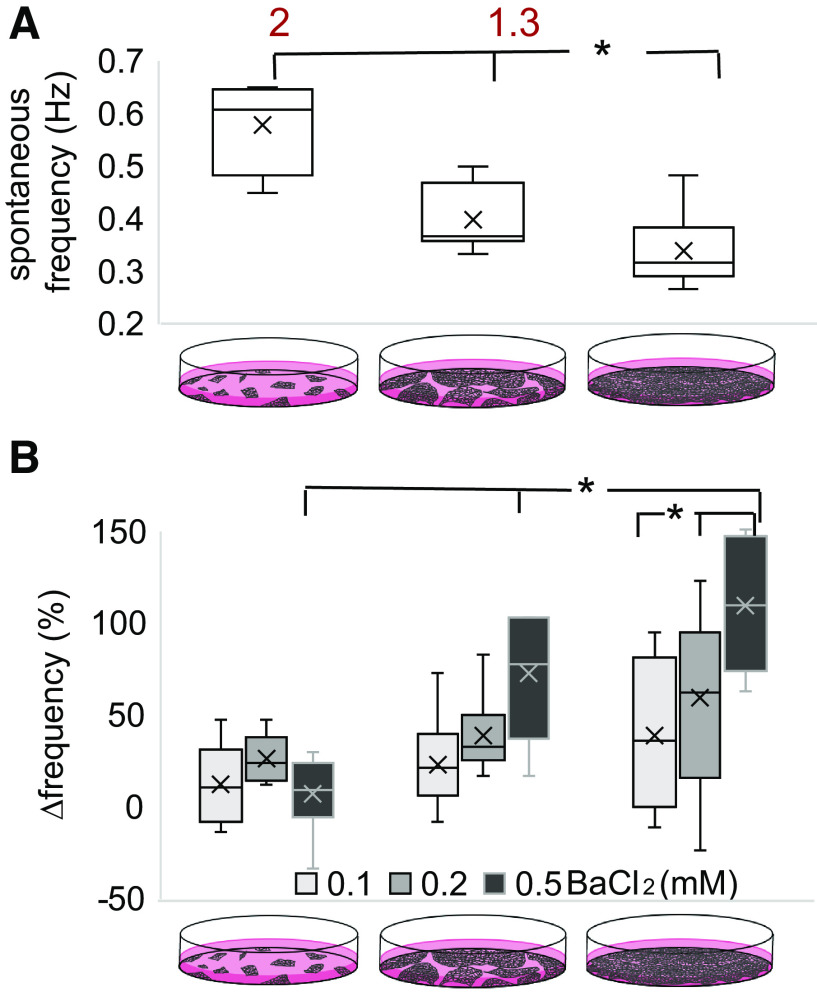
Unmasking the contributions of inward rectifier potassium current (*I*_k1_)-like current to spontaneous activity across different cell culture densities using BaCl_2_. *A*: spontaneous frequency (Hz) of human induced pluripotent stem cell-derived cardiomyocytes (hiPSC-CMs) decreases with the increase of cell culture density (*P < 0.05); 2-fold and 1.3 times higher frequency in low- and medium-density groups compared with high-density samples. *B*: pharmacological probing with different doses of BaCl_2_ and changes in spontaneous frequency (%) from baseline. The syncytial high-density group shows a dose-dependent increase in the frequency of oscillations, as expected from *I*_k1_ block (*P < 0.05). Effects of BaCl_2_ without clear dose dependence in low-density samples and weaker effects in medium-density samples are potentially indicative of minimal *I*_k1_. Data for this plot were from several locations within *n* = 6 multicellular samples (5 for the low-density group with several isolated regions), subjected to the full sequential drug treatment protocol (12 cases of density and drug dose), data are presented as box-whisker plot. Additional experiments with parallel (nonsequential) administration of BaCl_2_ doses were done with a different batch of cells, yielding similar outcomes, as presented in Supplemental Fig. S3.

Dual functional measurements of voltage and intracellular calcium (Fig. 1, *C* and *D*) were carried out in a 96-well plate format using all-optical methods, i.e., our OptoDyCE platform (22, 23). Quantification of spontaneous oscillations under different cell plating densities (Fig. 4*A*) revealed a decrease in spontaneous beating frequency with the increase of cell culture density, e.g., the low-density group had approximately twofold higher intrinsic frequency of oscillations compared with the high-density cultures. For the sparser cultures, we averaged the frequency of oscillations recorded at multiple clusters within a well.

To further unmask functional Kir2.1’s role in yielding different spontaneous frequency rates seen as cell density varies, we applied a classic pharmacological blocker of *I*_k1_ (BaCl_2_) to the samples from the three density groups. On the basis of earlier studies in human iPSC-CMs (6, 27, 42), we used concentrations of 0.1 to 0.5 mM BaCl_2_ to achieve selectivity. The effect on oscillatory activity was quantified as a relative change in spontaneous frequency (%) from untreated conditions, normalized to levels before drug application in the same samples. As summarized in Fig. 4*B* (experiments done according to the sequential dosing described in Fig. 1*D*), the syncytial high-density group showed a well-defined dose-dependent increase in the frequency of oscillations, as expected from blocking endogenous *I*_k1_-like current (*P < 0.05). In the middle- and low-density groups, such dose-dependent changes did not exist or were less pronounced, in line with the lower KCNJ2 transcription and Kir2.1 protein expression in Fig. 3. These findings were corroborated in alternative sets of experiments, where BaCl_2_ was applied in parallel to physically different samples (Supplemental Fig. S3.

#### Lower cell density and suppression of I_K1_ increase occurrences of irregular activity.

In addition to the increased frequency of spontaneous activity, the lower-density samples exhibited more irregular activity and variability in the morphology of the action potentials, as seen in other studies as well (10, 19, 21, 47). Irregular activity included *1*) small fluctuations during the resting membrane potential, *2*) action potentials with variable amplitude and duration, and *3*) embedded lower-frequency wave component in the records.

**Fig. 5.**
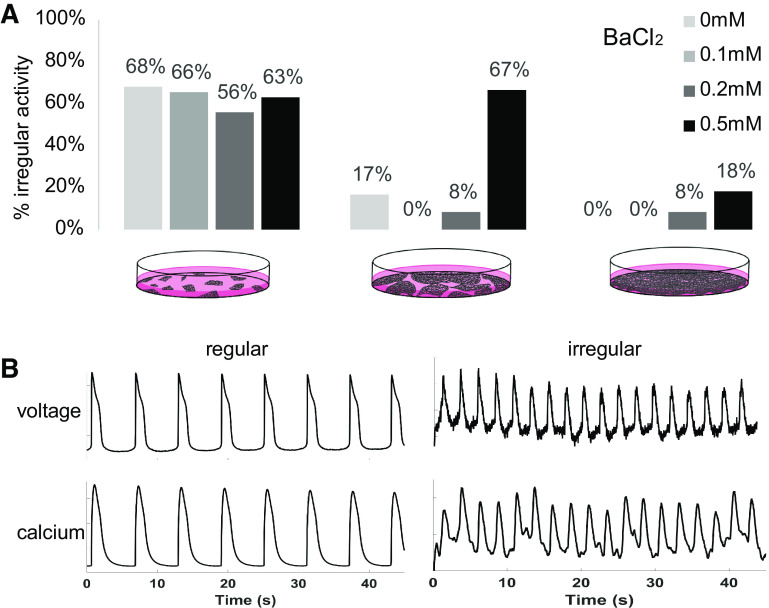
Irregular activity as function of cell density and suppression of inward rectifier potassium current (*I*_k1_)-like current by BaCl_2_. *A*: irregular activity is prominent in the low-density group at baseline (no BaCl_2_) and is brought about in medium- and high-density groups by high doses of BaCl_2_. *B*: example traces (spontaneous voltage and calcium) for regular and irregular activity. These results were from multiple records *n* = 11–29 per case (12 cases of density and drug dose); see Table of record numbers in the Supplemental Material.

To quantify irregular activity among the different cell density groups, membrane voltage and intracellular calcium were recorded optically. As summarized in Fig. 5, no irregular activity was observed in the analyzed traces from the middle- and high-density groups under control conditions, whereas 75% of the low-density cell samples contained such records. Increasing doses of BaCl_2_ to block *I*_k1_-like current brought about irregular activity in the middle- and high-density groups. Again, the high cell density group remained with the least percentage of irregular activity events but showed dose dependence. The specific action of BaCl_2_ on *I*_k1_ and the dose-response in the high-density group potentially support higher functional Kir2.1 contribution compared with lower-density samples.

#### In paced conditions, Kir2.1 levels mainly influence the action potential upstroke, with milder effects on APD.

Previous dynamic clamp studies and computational work have indicated effects of *I*_k1_ levels on the morphology of the action potentials in hiPSC-CMs (31). To study these in controlled conditions, we combined the optical records with optical pacing (23). The recorded paced action potentials (Supplemental Fig. S4; 0.6–0.8 Hz at room temperature) were analyzed by features like action potential upstroke velocity and action potential duration at 90% repolarization.

**Fig. 6.**
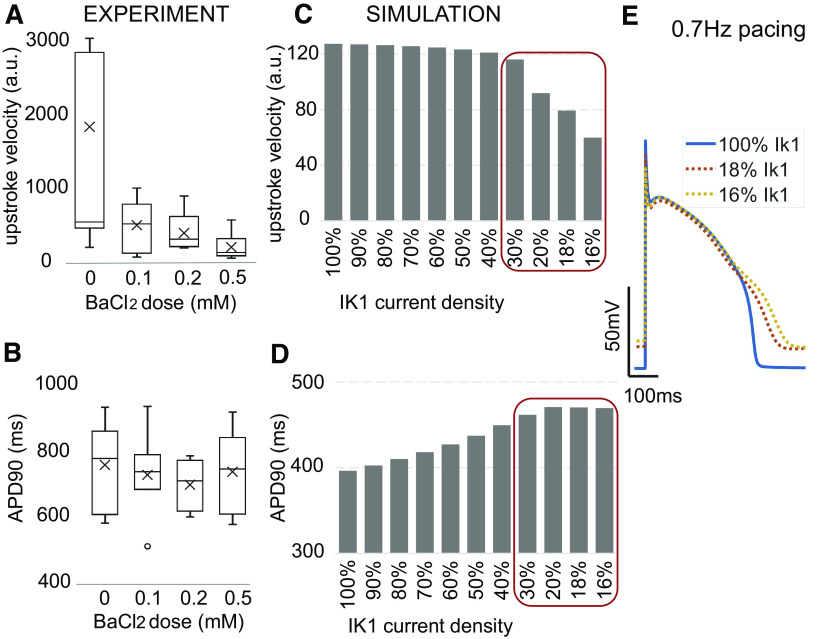
Effects of BaCl_2_ blocking of inward rectifier potassium current (*I*_k1_)-like current on optically paced action potentials in syncytium human induced pluripotent stem cell-derived cardiomyocytes (hiPSC-CMs) and computer-simulated blocking of *I*_K1_ in ventricular cells. *A*: experimentally obtained maximum upstroke velocity (a.u.) from voltage-sensitive fluorescence dye’s records (∆F/F) of optically paced action potentials, 0.7 Hz.*B*: action potential duration at 90% repolarization (APD_90_) under different BaCl_2_ doses. Upstroke velocity decreases as function of BaCl_2_ dose, whereas APD_90_ shows minor changes with BaCl_2_ treatment. Data are presented as box-whisker plot (*n* = 4 to 9 high-density multicellular samples for each of the drug doses; multiple locations averaged). *C*: computational results for upstroke velocity in adult ventricular cardiomyocytes as a function of *I*_k1_, analogous to *A*. *D*: APD_90_ in adult ventricular cardiomyocytes as a function of *I*_k1_, analogous to *B*. *E*: example traces of simulated action potentials paced at 0.7 Hz for different levels of *I*_k1_. Red boxes in *C* and *D* outline the relevant low-*I*_k1_ (<30%) regions of the plots as likely seen in the experimental hiPSC-CMs data in *A* and *B*, found to agree.

Blocking *I*_k1_-like current with increasing doses of BaCl_2_ in the high-density samples led to a dose-dependent decrease of AP upstroke velocity (Fig. 6*A*), likely due to lower availability of Na^+^ channels as a result of the more depolarized resting membrane potential. Negligible effects were observed on APD_90_ in the tested range of *I*_k1_ values (Fig. 6*B*). Although the functional experiments were done under room temperature instead of physiological temperature, computer simulations with a generic adult cardiac ventricular cell model (28) matched the experimental results considering expected low levels of Kir2.1 (under 30%, see red-outlined areas, Fig. 6, *C* and *D*). Note that the responses to blocking of *I*_k1_ were different if the cells expressed higher Kir2.1 levels. Similar effects were reported from a dynamic clamp study in hiPSC-CMs and computations (31), where *I*_k1_ injection varied from 1 to 10 pA/pF. Based on comparison with that study, the red-boxed range of relevant *I*_k1_ values, where AP upstroke is sensitive to *I*_k1_ block and APD_90_ is not sensitive, likely encompasses peak outward *I*_k1_ values below 3 pA/pF for our syncytial high-density hiPSC-CMs; this range includes the values for adult human cardiomyocytes.

**Fig. 7.**
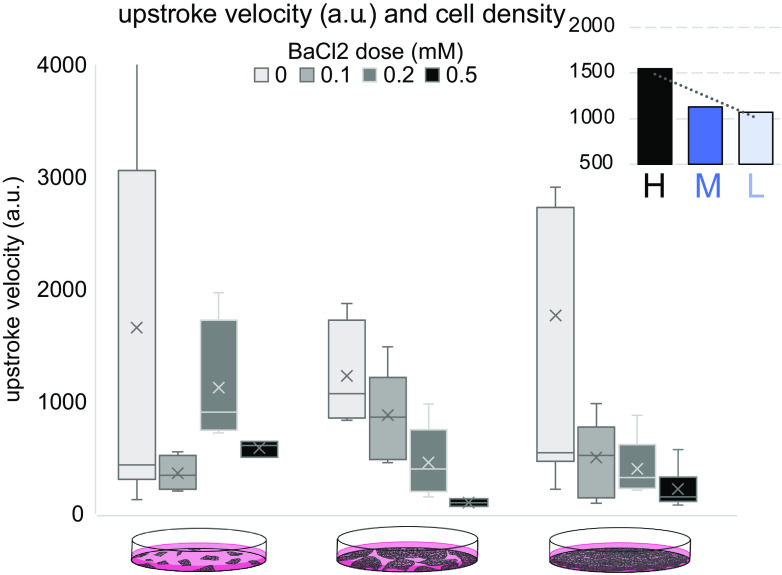
Sensitivity to BaCl_2_ blocking of inward rectifier potassium current (*I*_k1_)-like current as function of cell density. Dose response of experimentally obtained maximum upstroke velocity (a.u.) of optically paced action potentials when BaCl_2_ is applied. Effects vary between the high (H)-, middle (M)-, and low (L)-density syncytium human induced pluripotent stem cell-derived cardiomyocyte (hiPSC-CM) cultures. *Inset*: changes of means in upstroke of BaCl_2_ treated (0.5 mM) samples and control samples for the 3 cell densities, with the most pronounced seen in the high-density group. Data (*n* = 3–9 multicellular samples for each of the 12 cases of density and drug dose, multiple locations averaged) are shown as box-whisker plot.

Extending this to variations in cell density and dosing with BaCl_2_, we found that dose-dependent decrease trends for the maximum upstroke velocity exist in the high- and middle-density groups but not the low-density group (Fig. 7). When the decrease in maximum upstroke velocity before and after 0.5 mM BaCl_2_ treatment (Fig. 7, *inset*) is compared, we see the biggest difference in the high cell density group, corroborating Kir2.1 functional contribution in that group.

#### Cell density also influences responsiveness to blockers of spontaneous calcium release.

In addition to the *I*_k1_-mediated mechanism of spontaneous oscillations, activation of the forward mode of the Na^+^/Ca^2+^ exchanger and abnormal opening of the ryanodine receptors both can increase the probability for spontaneous calcium release (SCR) in cardiomyocytes (Supplemental Fig. S5*A*). If SCR contributes to oscillatory activity along with the low *I*_k1_ levels, blocking the function of either of the calcium-related proteins should decrease cell automaticity. Here, we find that different cell culture densities vary in their sensitivity to the blocking of these channels. As expected, the low-density cultures were found more sensitive to blockade of SCR because of their closeness to the excitation threshold, potentially due to lower *I*_k1_ (a key force in stabilizing the resting membrane potential). While 10 μM ryanodine caused only an ∼15% decrease of spontaneous activity in the high-density culture, the low-density group experienced stronger effects, in a greater change range. Blocking NCX by SEA0400 also had a higher effect in the low-density culture compared with the high-density culture. The SEA0400 dosage increase from 2 to 4 μM showed relative dose-dependent effects in both groups (Supplemental Fig. S5*B*).

## DISCUSSION

Applications of human iPSC cardiomyocytes for better understanding of human cardiac disease and for personalized medicine require their functional maturation (7, 13). In the native heart, cardiomyocytes are functioning as well-connected excitable units. Syncytial in vitro growth of hiPSC-CMs is expected to help create conditions that are closer to the native environment compared with isolated cells and therefore yield a more mature phenotype.

Studies of improving maturity of hiPSC-CMs over the past decade span a range of approaches, which include micropatterning, modifying the supporting/extracellular matrix to enhance focal-adhesion signaling (15), mechanical stretching, electrical field stimulation, induction of cell alignment, genetic manipulations to induce gene expression, delivery of biochemical factors, such as triiodothyronine and α-adrenergic agonist phenylephrine or a combination of these (48). While these strategies of various sophistication levels all may help the maturation of hiPSC-CMs, the fundamental cell-cell contact effects brought about by syncytial cell growth may represent a simple and impactful way to yield a more mature phenotype for hiPSC-CMs.

Here, we used protein quantification to establish the expression of Kir2.1 (the dominant ion channel correlate for *I*_K1_) in syncytia of hiPSC-CMs, albeit at ∼6 times lower levels than in adult human or rat heart tissue (Fig. 1). This is in contrast with prior studies in isolated hiPSC-CMs that found *I*_k1_ completely lacking (27, 42) or at negligible levels. Further pharmacological investigation (using dynasore as a blocker of clathrin-mediated internalization pathways; Supplemental Fig. S1) revealed that faster protein degradation is likely not the cause of the lower Kir2.1 protein levels in hiPSC-CMs compared with adult ventricular myocytes, and instead perhaps protein synthesis is different.

To assess the impact of cell density on the contribution of KCNJ2 gene transcription and Kir2.1 protein expression, we created conditions of variable cell density growth and probed KCNJ2 mRNA and Kir2.1 in two hiPS-CMs lines. When we reduced plating density from high by 60%, the corresponding reduction for the female and male cell lines was in the range of 30–39% for both mRNA (KCNJ2) and protein levels (Kir2.1). These results correlate with the level of functional changes in spontaneous rate, upstroke velocity during pacing, and irregular oscillations, all consistent with increased *I*_k1_-like current contribution in denser cell cultures(Figs. 4–7).

To confirm hiPSC-CMs culture densities’ effects on functional Kir2.1, all-optical electrophysiology was applied to probe spontaneous and optical paced cell activities with and without pharmacological perturbation. The highest-density syncytial samples showed the lowest frequency of spontaneous oscillations (as mature ventricular cells would) and the lowest occurrence of irregular rhythms (Figs. 4 and 5). Furthermore, pharmacological probing of the contributions of *I*_k1_-like current by variable doses of BaCl_2_ revealed a dose-response only in these high-density samples, whereas minimal effects were registered in the lower-density samples, with presumably missing *I*_k1_ (Figs. 4–7 and Supplemental Fig. S3).

Comparing experimental data on the effects of lower Kir2.1 levels for paced action potentials in the hiPSC-CMs with computational predictions for adult ventricular myocytes with variable levels of *I*_k1_, we identified key features that are sensitive to *I*_k1_ variations, AP upstroke in particular. Such computations also helped us narrow down the likely range of *I*_k1_ peak outward current in the syncytial hiPSC-CM samples to be below 3–4 pA/pF (Fig. 6), in line with some previous reports (31). Cell density was found to impact also alternative (to the *I*_k1_) potential mechanisms of spontaneous oscillations in the hiPSC-CMs, related to spontaneous calcium release; Supplemental Fig. S5). Frequency of oscillations was more easily suppressed by blockers of NCX and of the RyRs in the lower-density samples. Theoretically, in conditions of minimal *I*_k1_ and therefore relatively depolarized resting membrane potential, spontaneous calcium release is expected to more easily lead to voltage oscillations.

Overall, our results draw attention to the importance of syncytial conditions for the electrophysiology of hiPSC-CMs; they corroborate previous studies where cell growth density was shown to influence hiPSC-CMs’ action potential morphology (18, 47). Furthermore, earlier work with neonatal rat cardiomyocytes demonstrated cell density influence on the expression of potassium ion channels (14, 16) as well as general transcriptomics impact (47). Our results showing that cell density of hiPSC-CMs influences KCNJ2 transcription, Kir2.1 expression, and related functional contribution to hiPSC-CMs electrophysiology is in line with such studies.

A potential mechanism for these effects is the impact that cell-cell contacts have on the expression of ion channels, contributing to differences in the electrophysiology of isolated cells compared with that of cells within a syncytium. Colocalization of ion channels, for example the sodium channel Nav1.5 (encoded by SCN5a), with gap junctional structures (Cx43) has been demonstrated before in cardiac tissue (25). Macromolecular complexes of ion channels providing opposing ion fluxes at the cell membrane, e.g., Nav1.5 and Kir2.1, and their colocalization with the intercalated disks and the perinexus area of cell-cell contacts (32, 44), are of particular interest as an efficient mechanism for control of cardiac excitability. Such partnerships and several other known ion channel macromolecular assemblies (1) imply that Kir2.1 expression is indeed likely regulated by cell-cell contacts and ion channel clustering, driven by adhesion and gap-junctional proteins, which would exist only in multicellular preparations. This can explain and reconcile our results showing Kir2.1 contribution in hiPSC-CMs only in the denser cell cultures and previous patch-clamp studies reporting lack of *I*_k1_ in isolated cardiomyocytes.

It is important to point out that the classic tools to directly study cardiac electrophysiology, e.g., the patch clamp and voltage clamp, rely primarily on cells in isolation. The limitations of these classic approaches have been pointed out recently (18), specifically in the context of lower seal resistance and ability to assess resting membrane potential when *I*_k1_ levels are low. As in the study by Horvath et al. (18), which analyzed and compared data from isolated cells and from hiPSC-CM monolayers and 3D engineered tissues to find similarities between properties of multicellular hiPSC-CM samples and adult heart tissue, we highlight the importance of the syncytial setting for cardiomyocytes. Cell dissociation procedures are likely harsher on hiPSC-CM aggregates compared with human heart tissue.

All-optical cardiac electrophysiology, as used here, offers powerful ways to probe cell phenotypes within the multicellular setting. Yet, the approach used in this study comes with several limitations. For example, in its present form, it cannot directly report contributions of a specific ion current as voltage clamp does. However, this approach can provide a comprehensive view of functional responses of hiPSC-CMs in a syncytium and can do so in a contactless high-throughput manner (11, 12). From such high-content measurements, the contributions of a particular ion channel can be inferred indirectly. The observed functional changes in this study (lower density leading to higher spontaneous rates, more irregular activity, slower upstroke velocity, and little change in APD), along with computer modeling results for low *I*_k1_, the mRNA and protein quantification results under different cell density in different cell lines (male and female), taken together, suggest *I*_k1_-like contributions as a key possible factor. Further limitations include our measurements being done at room temperature instead of physiological temperature. To overcome the indirect nature of probing ion channels by all-optical electrophysiology, further developments may make it possible to also obtain presently missing quantitative ion current information by implementing versions of an optical clamp (35) in conjunction with computational tools. Furthermore, improved computer models or population of models of hiPSC-CMs (34), constrained by all-optical electrophysiology data, may be useful as in silico tools to further help interpret the effects of cell density and cell-cell coupling: a relevant feature that may provide an alternative explanation (to *I*_k1_ effects) for some of the results and was not independently probed in this study.

In summary, this study illustrates that syncytial growth of hiPSC-CMs represents a simple approach to influence ion channel expression toward a potentially more mature phenotype. All-optical electrophysiology techniques can help track the functional responses of hiPSC-CMs and accelerate their application to personalized medicine and optimization for heart regeneration purposes.

## GRANTS

This work was supported in part by National Heart, Lung, and Blood Institute Grant R01 HL-144157 and by grants from the National Science Foundation EFMA 1830941 and PFI 1827535 to E.E.

## DISCLOSURES

No conflicts of interest, financial or otherwise, are declared by the authors.

## AUTHOR CONTRIBUTIONS

W.L. and E.E. conceived and designed research; W.L. and J.L.H. performed experiments; W.L., J.L.H. and E.E. analyzed data; W.L. and E.E. interpreted results of experiments; W.L. and E.E. prepared figures; W.L. and E.E. drafted manuscript; W.L. and E.E. edited and revised manuscript; W.L., J.L.H. and E.E. approved final version of manuscript.
